# Role of Neuron-Specific Enolase in the Diagnosis and Disease Monitoring of Sarcoidosis

**DOI:** 10.1155/2022/3726395

**Published:** 2022-05-26

**Authors:** Noriaki Sunaga, Yasuhiko Koga, Yoshimasa Hachisu, Koichi Yamaguchi, Masaki Aikawa, Norimitsu Kasahara, Yosuke Miura, Hiroaki Tsurumaki, Masakiyo Yatomi, Reiko Sakurai, Toshitaka Maeno, Takeshi Hisada

**Affiliations:** ^1^Department of Respiratory Medicine, Gunma University Graduate School of Medicine, Maebashi, Japan; ^2^Innovative Medical Research Center, Gunma University Hospital, Maebashi, Japan; ^3^Oncology Center, Gunma University Hospital, Maebashi, Japan; ^4^Gunma University Graduate School of Health Sciences, Maebashi, Japan

## Abstract

Sarcoidosis is a systemic granulomatous disease of unknown etiology. The diagnosis of sarcoidosis is based on clinicopathologic findings accompanied by the formation of granulomas in multiple organs, including the lung. Although angiotensin-converting enzyme (ACE) and soluble interleukin 2 receptor (sIL-2R) are traditionally used for the diagnosis of sarcoidosis, specific diagnostic markers remain to be determined. In the current study, we found that serum neuron-specific enolase (NSE) levels were elevated in patients with sarcoidosis. Serum NSE levels were positively correlated with serum ACE and sIL-2R levels. The sensitivity of NSE alone was modest, but its combination with sIL-2R and ACE had the highest sensitivity compared to those of each single marker. When comparing serum NSE and pro-gastrin-releasing peptide (ProGRP) levels in SCLC patients with those in patients with sarcoidosis and nonsarcoidotic benign diseases, serum NSE could be used to distinguish SCLC from sarcoidosis and nonsarcoidosis by setting at a cutoff value of 17.0 ng/ml with a sensitivity of 73.5% and a specificity of 90.2%, which were comparable to those of ProGRP. Serum NSE levels were associated with organ involvement and were higher in sarcoidosis patients who had been treated with oral corticosteroid (OCS) than in those who had never received OCS therapies; there was a positive association between elevated serum NSE levels and OCS use. Increased concentrations of serum NSE in patients at the nonremission phase decreased after spontaneous remission, whereas serum NSE levels fluctuated in accordance with serum ACE or sIL-2R levels during the follow-up period in patients with sarcoidosis. These findings suggest that NSE could be a marker for the diagnosis and monitoring of the clinical outcome of patients with sarcoidosis.

## 1. Introduction

Sarcoidosis is a systemic granulomatous disease that involves multiple organs, including the lung, eyes, and skin [1]. Sarcoidosis lesions are histologically characterized by noncaseating granuloma formed by an exaggerated immune response originating from the interaction between macrophages and T cells [2]. The majority of patients with sarcoidosis are initially diagnosed with asymptomatic bilateral hilar lymphadenopathy (BHL) on chest radiography [1]. Thus, it is critical for the diagnosis to exclude other diseases showing hilar or mediastinal lymphadenopathy, such as small cell lung cancer (SCLC), which frequently involve lymph node metastases [3]. Although clinical laboratory testing of serum angiotensin-converting enzyme (ACE), lysozyme, neopterin, and soluble interleukin 2 receptor (sIL-2R), which are produced by activated T cells or macrophages, is used as a diagnostic marker for sarcoidosis, their diagnostic ability is not satisfactory [4–6]. Chitotriosidase has also been considered as a candidate biomarker for the progression of sarcoidosis, especially for the advanced pulmonary stage [7, 8]. However, chitotriosidase gene polymorphisms influence the expression of chitotriosidase [9], such as the insertion/deletion polymorphism of the *ACE* gene, which impairs the diagnostic value of ACE [10]. Therefore, it is worthwhile to search for novel diagnostic markers for sarcoidosis.

Neuron-specific enolase (NSE) is a neuron-specific glycolytic isozyme of enolase mainly expressed in neurons and neuroendocrine cells [11] and is well known as a tumor marker for SCLC [12]. NSE is also expressed in nonneural cells such as platelets, lymphocytes, and macrophages [11, 13]. Previous studies have shown that serum NSE levels increase in nonmalignant lung diseases such as pulmonary tuberculosis (TB) and pulmonary alveolar proteinosis [14–16]. Of note, serum NSE concentrations were related to the activity and severity of pulmonary TB, in which a large number of macrophages around the granulomatous lesions were stained with NSE [15]. In addition, previous studies reported that elevated serum NSE levels were associated with increased inflammatory states in kidney diseases [17] and Crohn's disease [18]. These observations suggest that serum NSE levels could be elevated in patients with nonmalignant pulmonary diseases accompanied by active inflammation, whereas there is no comprehensive study assessing the significance of serum NSE as a biomarker for sarcoidosis. In the present study, we retrospectively investigated serum NSE levels and their correlations with other traditional markers (ACE and sIL-2R) in patients with sarcoidosis and evaluated the significance of NSE for the diagnosis of sarcoidosis and monitoring of clinical outcomes.

## 2. Materials and Methods

### 2.1. Patients

We conducted a retrospective study of consecutive patients with sarcoidosis and nonsarcoidotic diseases who were assessed for an examination of serum NSE, ACE, sIL-2R, and pro-gastrin-releasing peptide (ProGRP) at the Gunma University Hospital between January 2005 and May 2020 (Table 1). Medical records were reviewed, and patients with sarcoidosis based on clinical findings, histological demonstration of noncaseating epithelioid cell granuloma, and exclusion of other diseases capable of producing a similar histological or clinical picture as recommended by the American Thoracic Society (ATS)/European Respiratory Society (ERS)/World Association of Sarcoidosis and Other Granulomatous Disorders (WASOG) statement were included in the present study [19]. Serum levels of NSE, ACE, sIL-2R, and ProGRP in patients who had been suspected of having malignant diseases, such as SCLC, in addition to sarcoidosis at the initial diagnosis were analyzed in this study. The characteristics of patients with nonsarcoidotic diseases are shown in Table S1. The data from the hemolytic samples that showed false increases in NSE levels were excluded. In 6 patients with sarcoidosis who had not received treatment with systemic steroids or immunosuppressants, data on the serum NSE levels were available at the time when the patients were in nonremission and spontaneous remission statuses. Spontaneous remission was defined as the complete disappearance of lesions on CT scan accompanied by an improvement in physical symptoms without medical treatment [20]. The demographic and clinicopathological characteristics of the patients were collected from patient medical records.

### 2.2. Measurement of Serum NSE, ACE, and sIL-2R Concentrations

Serum concentrations of NSE and ProGRP were measured using Elecsys^®^ NSE (Roche Diagnostics, Mannheim, Germany) and Elecsys^®^ ProGRP (Roche Diagnostics), respectively. Serum ACE levels were measured by a colorimetric method with the substrate p-hydroxyhippuryl-L-histidyl-L-leucine using an ACEcolor kit (Fujirebio Inc, Tokyo, Japan). Serum sIL-2R levels were measured by an enzyme-linked immunosorbent assay using the Cell-Free IL-2R Test Kit (T Cell Sciences Inc., Cambridge, MA, USA). According to the manufacturer's instructions, the upper limits of normal for NSE, ACE, sIL-2R, and ProGRP were 12.0 ng/ml, 21.4 IU/L, 482 U/ml, and 80 pg/ml, respectively.

### 2.3. Statistical Analysis

Differences between two independent groups were analyzed by the Mann–Whitney test, whereas differences between two paired groups were analyzed by the Wilcoxon test. Correlations between two groups were analyzed by the Pearson correlation coefficient. Two nominal variables were compared using Fisher's exact test. The statistical analyses described above were performed using GraphPad Prism 8 for Mac OS X (GraphPad Software, San Diego, CA, USA). To determine the predicted probabilities for combined biomarkers, receiver operating characteristic (ROC) analysis and logistic regression analysis were conducted using IBM SPSS Statistics 25 software (IBM, Tokyo, Japan) to evaluate the diagnostic values of the serum markers alone and in combination. *P* < 0.05 was considered significant.

## 3. Results

**Figure 1:**
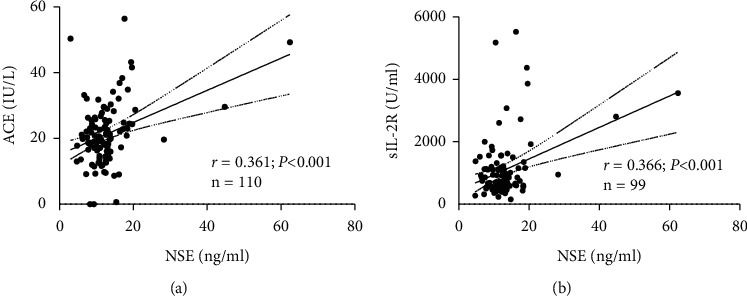
Significant correlations between (a) NSE and ACE and between (b) NSE and sIL-2R, as analyzed by the Pearson correlation coefficient. The best-fit line with its 95% confidence interval is shown.

We first investigated the serum levels of NSE, ACE, sIL-2R, and ProGRP in 114 patients with sarcoidosis. The median levels (ranges) of NSE, ACE, sIL-2R, and ProGRP were 12.1 (3.0–62.4) ng/ml, 20.2 (0–56.4) IU/L, 753 (157–5522) U/ml, and 42.5 (15.9–123.0) pg/ml, respectively. The correlations between serum NSE and the other markers ACE and sIL-2R were evaluated in patients who had undergone examinations of these markers at the same time of initial diagnosis. There were positive correlations between NSE and either ACE or sIL-2R (Figures 1(a) and 1(b)), whereas ProGRP was not significantly correlated with ACE or sIL-2R (data not shown). These results showed that serum NSE concentrations were increased with serum levels of ACE and sIL-2R in patients with sarcoidosis.

**Figure 2:**
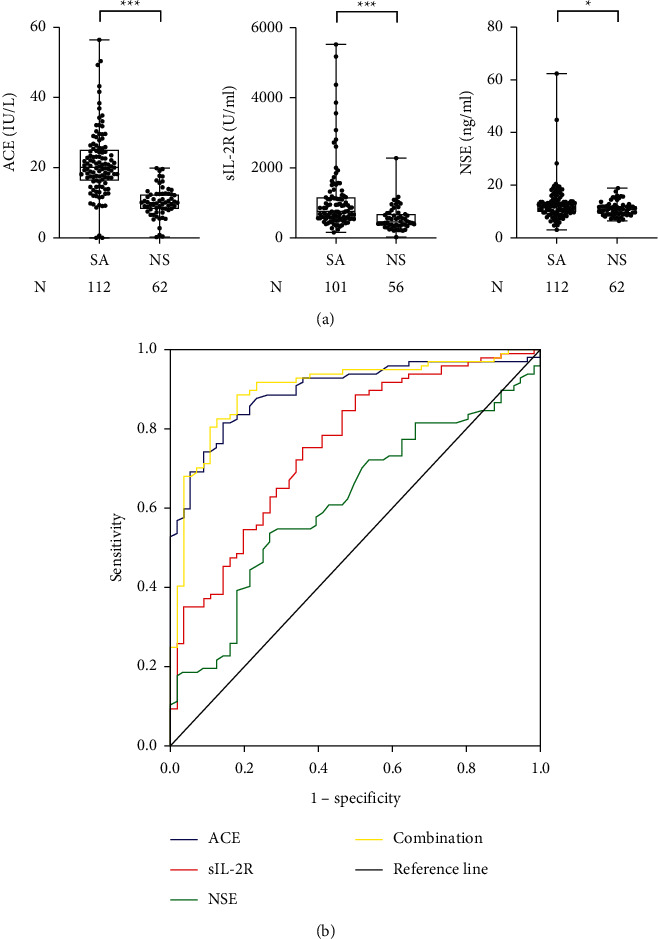
(a) Comparisons of serum levels of NSE, sIL-2R, and ACE in patients with sarcoidosis (SA) versus nonsarcoidotic benign diseases (NS). The box extends from the 25^th^ to 75^th^ percentiles with the line in the middle of the box indicating the median level. The whisker represents the minimum level to the maximum level. (b) Receiver operating characteristic (ROC) curves for NSE, ACE, sIL-2R, and their combination in patients with sarcoidosis. ^*∗*^*P* < 0.05; ^*∗∗∗*^*P* < 0.001.

When comparing the serum levels of these markers between patients with sarcoidosis and those with nonsarcoidotic benign diseases, the levels of NSE, ACE, and sIL-2R were significantly higher in the sarcoidosis group (Figure 2(a)). The sensitivity of serum NSE alone was modest, but its combination with sIL-2R (92.9%) and the combination of all three markers (93.8%) had higher sensitivities compared to those of each single marker at the cutoff values based on the manufacturer's instructions (Table 2). In addition, the negative predictive value of all three markers below their respective cutoffs (80.0%) was highest among the single markers and the combination. The ROC analysis revealed that the areas under the curve (AUCs) for NSE, ACE, sIL-2R, and the combination of these markers were 61.8%, 89.8%, 75.1%, and 90.0%, respectively (Figure 2(b); Table 3); the best discriminatory value of NSE was obtained at a cutoff point of 12.0 ng/ml, which was the same as that described in the manufacturer's instructions. On the other hand, the optimal cutoff value of ACE was 14.5 IU/L with a sensitivity of 78.6% and a specificity of 87.1%, whereas the optimal cutoff value of sIL-2R was 581 U/ml with a sensitivity of 71.3% and a specificity of 66.1% (Table S2).

Since NSE is a well-known tumor marker for SCLC, we compared serum NSE levels in SCLC patients with those in patients with sarcoidosis or nonsarcoidotic benign diseases. As expected, the SCLC patients exhibited significantly higher serum levels of NSE (median 27.2 ng/ml) and ProGRP (median 331.5 pg/ml) than the patients with sarcoidosis or nonsarcoidotic benign diseases. The ROC analysis showed that the AUCs for NSE and ProGRP were 86.7% and 85.5%, respectively (Figure S1; Table S3), and the optimal cutoff value of NSE was 17.0 ng/ml with a sensitivity of 73.5% and a specificity of 90.2%, which were similar to those of ProGRP in the present cohort including a large number of sarcoidosis patients (Table S4). Thus, serum NSE could be used to distinguish SCLC from sarcoidosis or nonsarcoidotic benign diseases by setting at a cutoff value of 17.0 ng/ml.

**Figure 3:**
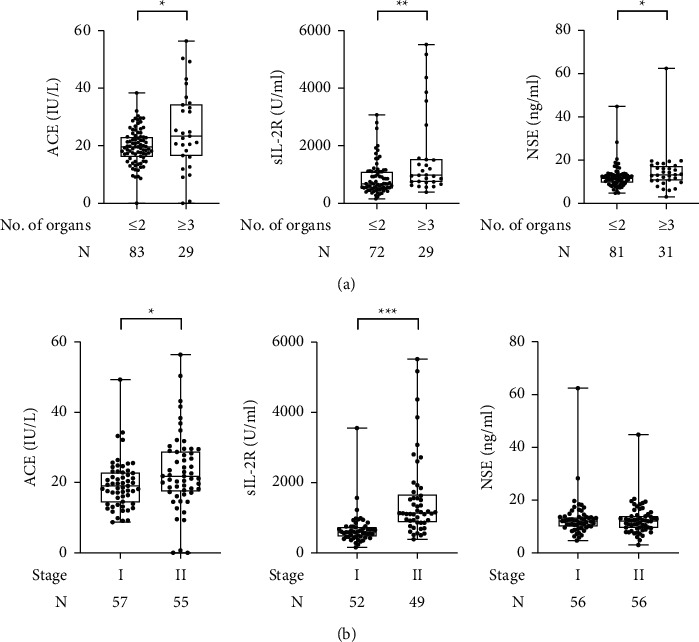
(a) Comparisons of the serum levels of NSE, sIL-2R, and ACE between sarcoidosis patients with ≤2 affected organs and those with ≥3 affected organs. (b) Comparisons of the serum levels of NSE, sIL-2R, and ACE between stage I sarcoidosis patients and stage II sarcoidosis patients. ^*∗*^*P* < 0.05^*∗∗*^*P* < 0.01; ^*∗∗∗*^*P* < 0.001.

To examine the association of serum NSE levels with the extent of disease in patients with sarcoidosis, the serum concentrations of NSE, ACE, and sIL-2R were compared according to the number of affected organs. The levels of these markers, including NSE, were significantly higher in sarcoidosis patients with 3 or more affected organs than in those with 2 or fewer affected organs (Figure 3(a)). Meanwhile, there was no significant difference in serum NSE levels between pulmonary stage I and stage II, unlike the results of ACE and sIL-2R (Figure 3(b)). Thus, it is likely that serum NSE increases as the number of involved organs increases, whereas the pulmonary stage of sarcoidosis may not be associated with serum NSE levels.

**Figure 4:**
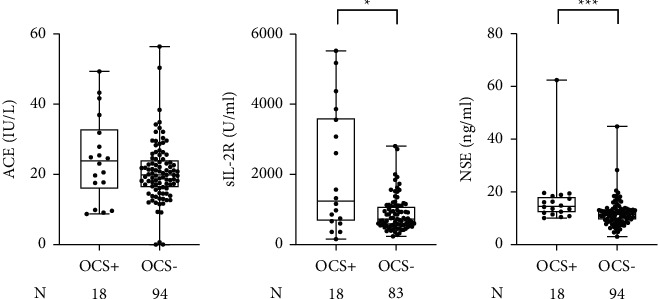
Comparisons of the serum levels of NSE, sIL-2R, and ACE between sarcoidosis patients who had been treated with OCS and those who had never been treated with OCS. ^*∗*^*P* < 0.05, ^*∗∗∗*^*P* < 0.001.

Given that NSE has been reported to reflect systemic inflammation [17, 18], we investigated whether high serum NSE levels were associated with oral corticosteroid (OCS) use in patients with sarcoidosis. Laboratory data of serum NSE, sIL-2R, and ACE were available for sarcoidosis patients who had received OCS therapy due to involvement of organs including the nervous system (*N* = 7), lung (*N* = 3), heart (*N* = 3), kidney (*N* = 3), skin (*N* = 2), and spleen (*N* = 1). Both serum NSE and sIL-2R levels, but not serum ACE levels, were significantly higher in sarcoidosis patients who had been treated with OCS than in those who had never received OCS therapies (Figure 4). When using the cutoff levels established by the manufacturer, there was a significant association between elevated serum NSE levels and OCS use with an odds ratio of 4.15 (Table 4). Alternatively, the use of OCS was not significantly associated with elevated levels of ACE or sIL-2R at either the cutoff values recommended by the manufacturer or the optimal cutoff values determined by the ROC analysis in this study (Table 4). These results indicate that elevated serum NSE levels could predict the likelihood of OCS use in patients with sarcoidosis.

**Figure 5:**
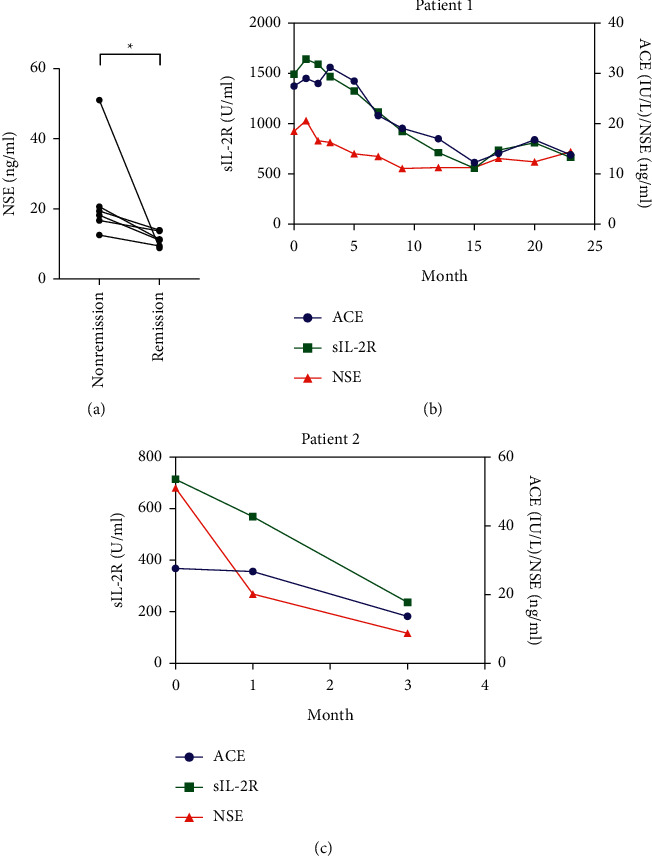
(a) Comparison of serum NSE levels between the remission and nonremission groups of 6 patients with sarcoidosis. Time courses of the laboratory data of ACE (IU/L), sIL-2R (U/ml), and NSE (ng/ml) in (b) Patient 1 and (c) Patient 2. ^*∗*^*P* < 0.05.

We further evaluated the changes in serum NSE levels during the follow-up period of sarcoidosis patients whose serum NSE values were available at the time of nonremission and spontaneous remission. The serum levels of NSE in the remission group significantly decreased compared with those in the nonremission group (Figure 5(a)). In addition, in patients whose laboratory data of serum ACE, sIL-2R, and NSE could be followed at multiple time points, the serum NSE levels appeared to fluctuate in accordance with the serum ACE or sIL-2R levels during the clinical course. In Patient 1, who was followed for more than 20 months with no systemic steroid therapy or immunosuppressants, changes in the levels of serum NSE, ACE, and sIL-2R significantly correlated with each other (Figure 5(b)). Patient 2 had Löfgren syndrome with high fever, polyarthralgia, erythema nodosum, and BHL, and his NSE level was high (51.0 ng/ml) at the time of initial diagnosis (Figure 5(c)). Three months later, the serum NSE level markedly decreased to a concentration of 8.8 ng/ml with spontaneous improvement in his symptoms and shrinkage of the mediastinal and hilar lymph nodes. These findings indicate that serum NSE is a useful marker for monitoring the sarcoidosis activity.

## 4. Discussion

Patients with sarcoidosis commonly exhibit asymptomatic thoracic lymphadenopathy, and various laboratory examinations are required for a differential diagnosis, including assessments for SCLC. Indeed, the patients who were assessed in this study had undergone laboratory examination of serum NSE, a well-known tumor marker for SCLC, because most of them had been suspected of having SCLC based on the radiological findings of mediastinal and hilar lymphadenopathy. We found that the serum NSE level was elevated in patients with sarcoidosis and positively correlated with the levels of other traditional markers, including ACE and sIL-2R, suggesting that NSE is a diagnostic marker for sarcoidosis. Consistent with our findings, an upregulated level of serum NSE was observed in a case report of a patient with sarcoidosis with multiple mediastinal and bilateral hilar lymphadenopathies who had been misdiagnosed with malignant tumors [21]. In another case report, an increased level of serum NSE was detected in a patient with necrotizing sarcoid granulomatosis [22]. Considering that NSE is expressed in macrophages [11, 13], it is plausible that serum NSE concentrations increase as a result of granuloma formation in sarcoidosis. This idea is supported by a previous study demonstrating that high levels of serum NSE were observed in TB patients who had high numbers of NSE-stained macrophages in granulomatous lesions [15].

There has been a persistent need for useful diagnostic markers for sarcoidosis because clinicians generally experience difficulty in diagnosing patients with sarcoidosis that affects multiple organs. ACE and lysozyme have been traditionally used as diagnostic markers for sarcoidosis, but their sensitivities are not satisfactory, ranging from 29% to 63% for ACE and from 26% to 79% for lysozyme [4, 23–27]. Moreover, the insertion/deletion polymorphism of the *ACE* gene influences its activity, thus impairing its diagnostic value [10]. Recently, sIL-2R and chitotriosidase have been shown to be superior diagnostic markers, with a sensitivity of 53–88% for sIL-2R [24, 28] and a sensitivity of 83–89% for chitotriosidase [7, 8]. However, chitotriosidase gene polymorphisms influence the expression of chitotriosidase, thus possibly causing false-negatives [9]. In our study, the sensitivity of NSE alone seems comparable to those of the traditional markers ACE and lysozyme, but its combinations with ACE and sIL-2R resulted in a greater sensitivity, providing the additional value of NSE in combination with such traditional markers for sarcoidosis. The prominent negative predictive value of triple-negative NSE, ACE, and sIL-2R also suggests that the combination of these markers is useful for excluding sarcoidosis during diagnosis. Thus, the examination of serum NSE may be prudent for diagnosing sarcoidosis.

We found that an increased number of affected organs was associated with elevated serum NSE levels, suggesting that NSE reflects the extent of active granulomatous lesions, as do ACE and sIL-2R, in patients with sarcoidosis. Previous studies have shown that serum levels of ACE and sIL-2R have a tendency to increase in patients with active sarcoidosis and are associated with the number of involved organs [20, 28–31]. Furthermore, the present study demonstrated that elevated serum levels of NSE, but not ACE or sIL-2R, were significantly correlated with OCS use in sarcoidosis patients, which is consistent with previous findings that elevated levels of NSE have been related to increased systemic inflammation [17, 18]. Our study also showed that serum NSE levels significantly decreased after spontaneous remission of sarcoidosis and that serum NSE levels fluctuated along with serum ACE and sIL-2R levels during the clinical course in some patients. Similarly, a previous study found that serum sIL-2R levels were significantly lower in spontaneous remission patients than in nonremission patients [20]. Although further investigation with a larger number of patients is needed, the present results indicate that NSE has a predictive role in the use of OCS and the clinical outcome of sarcoidosis.

In summary, the current study demonstrated that serum NSE concentrations were elevated in patients with sarcoidosis. The inclusion of NSE in combination with ACE and sIL-2R improved the sensitivity and negative predictive value for the diagnosis of sarcoidosis compared with each marker alone. The increased NSE levels were associated with the extent of organ involvement and OCS use and changed in accordance with the patients' disease status, indicating that NSE may be useful for predicting the use of OCS and monitoring sarcoidosis status. In clinical practice, serum NSE concentrations should be examined when SCLCs are suspected based on the radiological findings of bilateral and multiple mediastinal lymphadenopathies and pulmonary nodules. Our findings provide novel insight that NSE serves not only as a tumor marker but also as a diagnostic marker for sarcoidosis. Since the present findings were obtained from a cohort at a single institution, further investigation in another cohort will verify the role of NSE in the diagnosis of sarcoidosis.

## Figures and Tables

**Table 1:** Characteristics of patients in the present study.

Disease	Characteristic	Number	(%)
Sarcoidosis		114	
	Gender		
	Male	36	(31.6)
	Female	78	(68.4)
	Median age (range)	61	(14–86)
	Smoking status		
	Smoker	37	(32.5)
	Nonsmoker	77	(67.5)
	Number of involved organs		
	1	14	(12.3)
	2	69	(60.5)
	3	21	(18.4)
	4	10	(8.8)
	Type of involved organs		
	Lung	113	(99.1)
	Eye	81	(71.1)
	Skin	29	(25.4)
	Heart	13	(11.9)
	Nervous system	9	(7.9)
	Kidney	5	(4.4)
	Joint	2	(1.8)
	Spleen	1	(0.9)
	Chest radiographic stage		
	1	57	(50.0)
	2	56	(49.1)
	≥3	0	(0)
	Positive biopsy	75	(65.8)
	Oral corticosteroid therapy	19	(16.7)
Nonsarcoidotic benign diseases		62	
Small cell lung cancer		68	

**Table 2:** Diagnostic significance of ACE, sIL-2R, NSE, and the combination for sarcoidosis.

Parameter	No. of patients with sarcoidosis/nonsarcoidosis	*P* value	Sensitivity (%)	Specificity (%)	PPV (%)	NPV (%)
ACE (+)	46/0	<0.001	41.1	100	100	48.4
ACE (−)	66/62					
sIL-2R (+)	85/26	<0.001	84.2	53.6	76.6	65.2
sIL-2R (−)	16/30					
NSE (+)	57/16	0.001	50.9	74.2	78.1	45.5
NSE (−)	55/46					
ACE (+) and/or sIL-2R (+)	86/26	<0.001	86.9	53.6	76.8	69.8
Double (−)	13/30					
ACE (+) and/or NSE (+)	73/16	<0.001	66.4	74.2	82.0	55.4
Double (−)	37/46					
sIL-2R (+) and/or NSE (+)	92/32	<0.001	92.9	42.9	74.2	77.4
Double (−)	7/24					
ACE (+) and/or sIL-2R (+) and/or NSE (+)	91/32	<0.001	93.8	42.9	74.0	80.0
Triple (−)	6/24					

PPV: positive predictive value; NPV: negative predictive value. The data were obtained when cutoff values of ACE, sIL-2R. and NSE were 21.4 IU/L, 482 U/ml, and 12.0 ng/ml, respectively.

**Table 3:** ROC analysis for ACE, sIL-2R, NSE, and the combination for sarcoidosis.

Parameter	AUC	95% CI	*P* value
ACE	0.898	0.848–0.948	<0.001
sIL-2R	0.751	0.672–0.831	<0.001
NSE	0.618	0.527–0.709	0.017
Combination	0.900	0.847–0.953	<0.001

ROC: receiver operating characteristic; AUC: area under curve; CI: confidence interval.

**Table 4:** Likelyhood of oral corticosteroid use based on serum ACE, sIL-2R, and NSE in sarcoidosis.

Parameter	Cutoff value	No. of OCS-treated cases/OCS-untreated cases	Odds ratio	95% CI	*P* value
ACE (+) *vs.* ACE (−)	21.4	10/36 *vs.* 8/58	2.01	0.78–5.73	0.198
	14.5	14/74 *vs.* 4/20	0.95	0.30–2.87	>0.999
sIL-2R (+) *vs*. sIL-2R (−)	482	15/70 *vs.* 3/13	0.93	0.23–3.37	>0.999
	581	15/57 *vs.* 3/26	2.28	0.66–7.89	0.262
NSE (+) *vs.* NSE (−)	12.0	14/43 *vs.* 4/51	4.15	1.25–12.13	0.019

OCS: oral corticosteroid; CI: confidence interval.

## Data Availability

The data that support the findings of this study are available from the corresponding author upon reasonable request.

## Supplementary Materials

Supplementary Materials Table S1: characteristics of nonsarcoidotic patients in the present study. Table S2: diagnostic significance of ACE and sIL-2R for sarcoidosis at different cutoff points. Table S3: ROC analysis for NSE and ProGRP for diagnosis between SCLC and benign diseases. Table S4: cutoff value setting of NSE and ProGRP for differential diagnosis between SCLC and benign diseases. Figure S1: ROC curves for NSE and ProGRP in patients with SCLC and benign diseases (sarcoidosis plus nonsarcoidotic diseases).
