# Supplementary material for: Role of Neuron-Specific Enolase in the Diagnosis and Disease Monitoring of Sarcoidosis
Source: Can Respir J. 2022 May 26;2022:3726395. doi: 10.1155/2022/3726395 (PMC9162870; doi:10.1155/2022/3726395)
Supplement: Supplementary Materials — Table S1: characteristics of nonsarcoidotic patients in the present study. Table S2: diagnostic significance of ACE and sIL-2R for sarcoidosis at different cutoff points. Table S3: ROC analysis for NSE and ProGRP for diagnosis between SCLC and benign diseases. Table S4: cutoff value setting of NSE and ProGRP for differential diagnosis between SCLC and benign diseases. Figure S1: ROC curves for NSE and ProGRP in patients with SCLC and benign diseases (sarcoidosis plus nonsarcoidotic diseases). [file 3726395.f1.pdf]

Table S1. Characteristics of nonsarcoidotic patients in the present study.

Disease	Number
Nonsarcoidotic benign diseases	62
Idiopathic interstitial pneumonia	37
IPF	17
NSIP	12
COP	5
PPFE	3
Nontuberculous mycobacteria	5
Tuberculous lymphadenitis	2
Bacterial pneumonia	4
Other diseases	14
Pulmonary aspergillus	1
Chronic bronchitis	2
Nonspecific lymphadenopathy	2
Bronchial asthma	1
Spinal dural arteriovenous aneurysm	1
Spinal stenosis	2
Type 2 diabetes mellitus	1
Trigeminal paralysis	1
Degenerative spondylosis	3
Small cell lung cancer	68

Table S2. Diagnostic significance of ACE and sIL-2R for sarcoidosis at the different cutoff points.

Parameter	Cutoff value	No. of SA/NS	*P*-value	Sensitivity (%)	Specificity (%)	PPV (%)	NPV (%)
ACE	>21.4 IU/L	46/0	<0.001	41.1	100	100	48.4
	≤21.4 IU/L	66/62					
ACE	>14.5 IU/L	88/8	<0.001	78.6	87.1	91.7	69.2
	≤14.5 IU/L	24/54					
sIL-2R	>482 U/ml	85/26	<0.001	84.2	53.6	76.6	65.2
	≤482 U/ml	16/30					
sIL-2R	>581 U/ml	72/19	<0.001	71.3	66.1	79.1	56.1
	≤581 U/ml	29/37					

SA: sarcoidosis; NS: nonsarcoidosis; PPV: positive predictive value; NPV: negative predictive value.

Table S3. ROC analysis for NSE and ProGRP for diagnosis between SCLC and benign diseases.

Parameter	AUC	95% CI	*P*-value
NSE	0.867	0.806–0.927	<0.001
ProGRP	0.855	0.787–0.923	<0.001

SCLC: small cell lung cancer; ROC: receiver operating characteristic; AUC: area under curve; CI: confidence interval.

Table S4. Cutoff value setting of NSE and ProGRP for differential diagnosis between SCLC and benign diseases.

Parameter	Cutoff value	No. of SCLC/benign	*P*-value	Sensitivity (%)	Specificity (%)	PPV (%)	NPV (%)
NSE	>12 ng/ml	58/73	<0.001	85.3	58.1	44.3	91.0
	≤12 ng/ml	10/101					
NSE	>17 ng/ml	50/17	<0.001	73.5	90.2	74.6	89.7
	≤17 ng/ml	18/157					
ProGRP	>80 pg/ml	47/9	<0.001	73.4	89.2	83.9	81.3
	≤80 pg/ml	17/74					

SCLC: small cell lung cancer; PPV: positive predictive value; NPV: negative predictive value.

Fig. S1

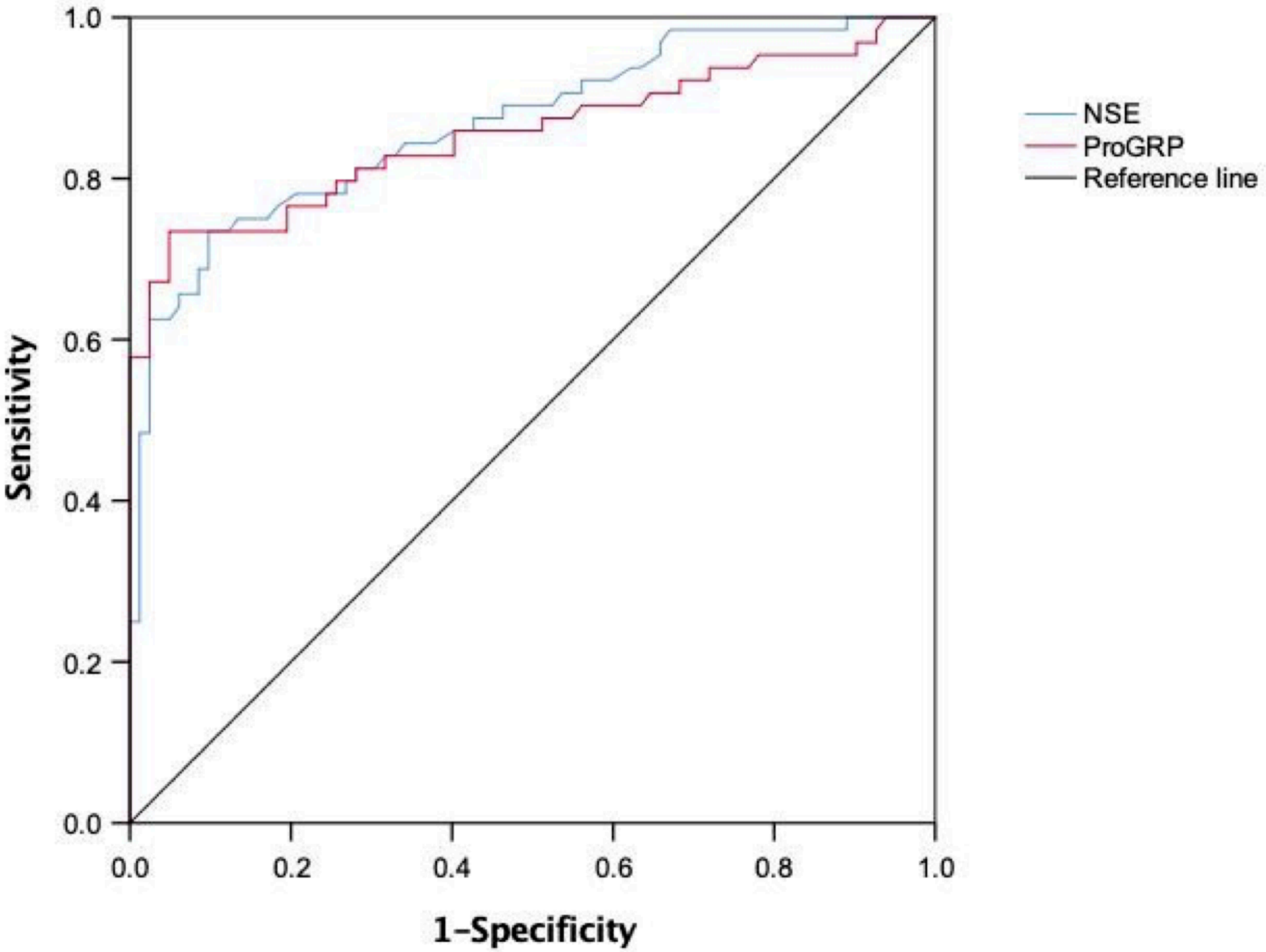

1.0
0.8
0.6
0.4
0.2
0.0
Sensitivity
0.0
0.2
0.4
0.6
0.8
1.0
1-Specificity
NSE
ProGRP
Reference line

Supplementary Figure Legends

Figure S1. ROC curves for NSE and ProGRP in patients with SCLC and benign diseases (sarcoidosis plus nonsarcoidotic diseases).
